# Ferruginol exhibits anticancer effects in OVCAR-3 human ovary cancer cells by inducing apoptosis, inhibition of cancer cell migration and G2/M phase cell cycle arrest

**DOI:** 10.3892/mmr.2021.11868

**Published:** 2021-01-26

**Authors:** Wen-Dong Xiong, Jian Gong, Chao Xing

Mol Med Rep 16: 7013-7017, 2017; DOI: 10.3892/mmr.2017.7484

Following the publication of the above paper, an interested reader drew to our attention that a number of apparent anomalies existed with the data presented in a couple of the figures in the above paper. Specifically, there appeared to be strikingly similar and duplicated patternings of cells within the cellular images featured in Figs. 3 and 4, which showed apoptotic induction as evaluated by fluorescence microscopy and TEM evaluation of ferruginol-induced apoptosis in OVCAR-3 human ovary cancer cells, respectively. Following an internal enquiry, the Editor of *Molecular Medicine Reports* has been able to verify the claims made by the interested reader; therefore, in view of the potential anomalies that have been identified and owing to a lack of overall confidence in the presented data, the Editorial Board have decided to retract the above paper from the publication. After having passed on this decision to the authors, they were in agreement that the paper should be retracted. The Editor apologizes to the readership of the Journal for any inconvenience caused.

